# Supplementary material for: Causal Relationship between Aging and Anorexia Nervosa: A White-Matter-Microstructure-Mediated Mendelian Randomization Analysis
Source: Biomedicines. 2024 Aug 16;12(8):1874. doi: 10.3390/biomedicines12081874 (PMC11351342; doi:10.3390/biomedicines12081874)
Supplement: Supplementary file 1 [file biomedicines-12-01874-s001.zip › Supplementary Figure/Figure S4/Singe SNP plot of MR analysis for TL to ubm-b-1998.pdf]

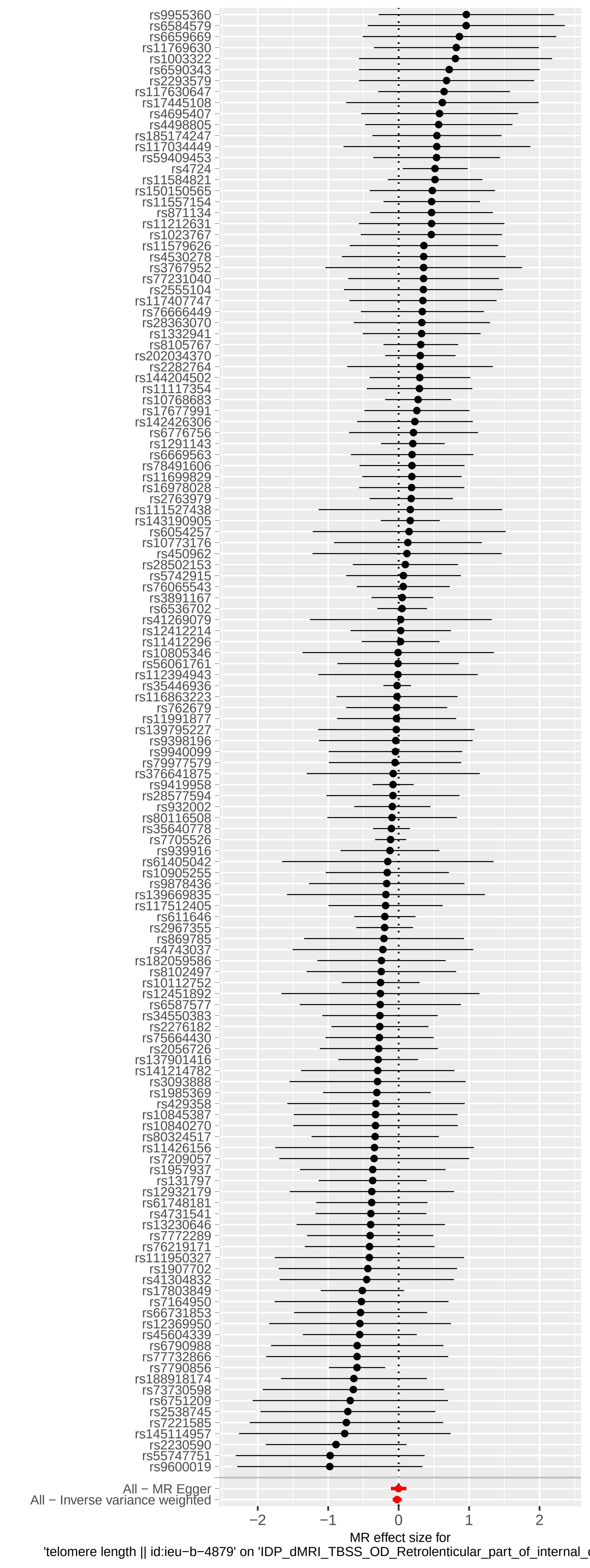

rs9955360
rs6584579
rs6659669
rs11769630
rs1003322
rs6590343
rs2293579
rs117630647
rs17445108
rs4695407
rs4498805
rs185174247
rs117034449
rs59409453
rs4724
rs11584821
rs150150565
rs11557154
rs871134
rs11212631
rs1023767
rs11579626
rs4530278
rs3767952
rs77231040
rs2555104
rs117407747
rs76666449
rs28363070
rs1332941
rs8105767
rs202034370
rs2282764
rs144204502
rs11117354
rs10768683
rs17677991
rs142426306
rs6776756
rs1291143
rs6669563
rs78491606
rs11699829
rs16978028
rs2763979
rs111527438
rs143190905
rs6054257
rs10773176
rs450962
rs28502153
rs5742915
rs76065543
rs3891167
rs6536702
rs41269079
rs12412214
rs11412296
rs10805346
rs56061761
rs112394943
rs35446936
rs116863223
rs762679
rs11991877
rs139795227
rs9398196
rs9940099
rs79977579
rs376641875
rs9419958
rs28577594
rs932002
rs80116508
rs35640778
rs7705526
rs939916
rs61405042
rs10905255
rs9878436
rs139669835
rs117512405
rs611646
rs2967355
rs869785
rs4743037
rs182059586
rs8102497
rs10112752
rs12451892
rs6587577
rs34550383
rs2276182
rs75664430
rs2056726
rs137901416
rs141214782
rs3093888
rs1985369
rs429358
rs10845387
rs10840270
rs80324517
rs11426156
rs7209057
rs1957937
rs131797
rs12932179
rs61748181
rs4731541
rs13230646
rs7772289
rs76219171
rs111950327
rs1907702
rs41304832
rs17803849
rs7164950
rs66731853
rs12369950
rs45604339
rs6790988
rs77732866
rs7790856
rs188918174
rs73730598
rs6751209
rs2538745
rs7221585
rs145114957
rs2230590
rs55747751
rs9600019
All − MR Egger
All − Inverse variance weighted
−2
−1
0
1
2
MR effect size for
'telomere length || id:ieu−b−4879' on 'IDP_dMRI_TBSS_OD_Retrolenticular_part_of_internal_c
